# An Audiovisual Correlation Matching Method Based on Fine-Grained Emotion and Feature Fusion

**DOI:** 10.3390/s24175681

**Published:** 2024-08-31

**Authors:** Zhibin Su, Yiming Feng, Jinyu Liu, Jing Peng, Wei Jiang, Jingyu Liu

**Affiliations:** 1 State Key Laboratory of Media Convergence and Communication, Beijing 100024, China; zhibin_su@163.com (Z.S.); jw@cuc.edu.cn (W.J.); 2 Key Laboratory of Acoustic Visual Technology and Intelligent Control System, Ministry of Culture and Tourism, Beijing 100024, China; fyming8888@163.com (Y.F.); ljy112233741@gmail.com (J.L.); 3 School of Information and Communication Engineering, Communication University of China, Beijing 100024, China; peggy202307@outlook.com

**Keywords:** fine-grained affects, music–video matching, audiovisual association, CCA feature fusion, factor analysis, hybrid matching model, affective similarity

## Abstract

Most existing intelligent editing tools for music and video rely on the cross-modal matching technology of the affective consistency or the similarity of feature representations. However, these methods are not fully applicable to complex audiovisual matching scenarios, resulting in low matching accuracy and suboptimal audience perceptual effects due to ambiguous matching rules and associated factors. To address these limitations, this paper focuses on both the similarity and integration of affective distribution for the artistic audiovisual works of movie and television video and music. Based on the rich emotional perception elements, we propose a hybrid matching model based on feature canonical correlation analysis (CCA) and fine-grained affective similarity. The model refines KCCA fusion features by analyzing both matched and unmatched music–video pairs. Subsequently, the model employs XGBoost to predict relevance and to compute similarity by considering fine-grained affective semantic distance as well as affective factor distance. Ultimately, the matching prediction values are obtained through weight allocation. Experimental results on a self-built dataset demonstrate that the proposed affective matching model balances feature parameters and affective semantic cognitions, yielding relatively high prediction accuracy and better subjective experience of audiovisual association. This paper is crucial for exploring the affective association mechanisms of audiovisual objects from a sensory perspective and improving related intelligent tools, thereby offering a novel technical approach to retrieval and matching in music–video editing.

## 1. Introduction

Modern sensor technology extends beyond the collection and enhancement of external signals to include the simulation of human audiovisual perception and cognitive capabilities. This encompasses tasks such as target classification [1,2], state prediction [3,4], public opinion monitoring [5], and emotional analysis [6]. For machines, the recognition and analysis of emotions represent an advanced perceptual task with a broad range of potential applications. Furthermore, audiovisual information serves as a crucial carrier for the deep exploration of emotional expression. Over the last decade, numerous intelligent algorithms and models have been developed to address these challenges. These models have been applied to unimodal [7] or multimodal [8] affective recognition tasks, yielding favorable identification outcomes. As the application domain expands, some research has turned its focus to intermodal correlation retrieval tasks [9]. However, compared to tasks that directly obtain recognition results, cross-modal matching methods aimed at audiovisual associations often exhibit lower accuracy [1], which is attributed to the absence of effective theoretical mechanisms to support these associations.

In the domain of music and video matching, various methods have been developed, including content-based matching [10], rhythm-based matching [11], and art style-based matching [12]. These methods contribute to achieving effective matching and fusion between audio and video. They primarily focus on the technical and stylistic features of music videos. However, it is discovered that emotion is indispensable for matching. The term “affective computing”, introduced by Picard, refers to all computational processes related to affect [13]. Matching music that aligns with the affective semantics expressed in the video can enhance affective expression in both modalities.

Some studies have explored audiovisual correlations based on a single emotional label. For example, researchers have conducted emotion recognition studies on videos or images first, followed by audio, and ultimately sought commonalities in emotional expression between the two [14]. However, this approach has not yielded satisfactory results. From a cognitive perspective, human emotional understanding is a complex process. Particularly in the study of fine-grained emotions, which allows for a more nuanced depiction of the complexities and subtle variations in emotions, it is imperative to delve deeper into the interrelations between various sensory modalities, rather than solely depending on the uniformity of labels or the similarity of features.

Complex affective expressions are often embedded within audiovisual works related to art. The emotion in artistic works is not presented in a single form, but tends to be the comprehensive expression of multiple emotions. Existing sentiment analysis methods can only provide coarse-grained categories of emotions [14,15,16] or measure arousal levels on emotional dimensions [15], such as the valence–arousal emotion model and the PAD emotion model. However, due to the limited expressive range of these emotion description models, it becomes challenging to conduct a more profound analysis of the matching mechanism and to obtain matching results that align with the psychological needs of viewers. Therefore, introducing fine-grained emotional expression is an effective problem-solving method in some complex audiovisual information perception scenarios.

Although researchers have made continuous progress in the task of audio–video cross-modal matching, visual information and auditory information inherently belong to different modalities. The emotional fusion mechanism between music and video has not been well explored. Thus, the emotion fusion mechanism of audiovisual works remains a long-term challenging task.

In summary, previous research and methods still have many shortcomings, including insufficient dimensions in affective classification, inadequate extraction of affective representation from features, and low accuracy in cross-modal matching between music and video. Therefore, the current research focus is on how to more accurately analyze and match audiovisual content at the affective level.

In this paper, we approach the problem from an affective perspective. Firstly, we extract feature vectors highly related to emotions from audio and video and establish a common subspace model using canonical correlation analysis (CCA) to fuse cross-modal features, thereby building an affective matching model at the feature level. Secondly, we select matching music–video pairs, divide them into multidimensional affective annotation words, and construct an affective space model at the factor level. This study not only contributes to applications such as music recommendation and video production but also holds significant value and practical implications in the promotion and publicity aspects of the film and television industry.

In order to solve these problems and further improve the research effect, the main research work and contributions in this paper are as follows:

1. A video and music dataset with fine-grained annotations of emotional arousal levels was constructed, with corresponding affective space for semantic analysis.
2. An extraction method for audiovisual affective features in film and television scenes was proposed. By distinguishing between samples with high matching degrees and those with low matching degrees, optimal correlation features were obtained based on the CCA method.
3. An audiovisual emotion matching model is proposed, which integrates the association of cross-modal features and similarity judgment of fine-grained emotions, enhancing the rationality of prediction results.

The paper is organized as follows. Section 2 discusses related works. In Section 3, the proposed method is presented. Next, Section 4 contains the experimental results and discussion. Finally, Section 5 reports on conclusions and future work.

## 2. Related Works

A few recent works on audio and video matching methods are reviewed below. Further, the work of affective music content analyses and feature extraction, affective video content analyses and feature extraction, as well as cross-modal retrieval and matching are also discussed in the sections below.

### 2.1. Audiovisual Affective Feature Extraction

In music emotion recognition (MER), the extraction of musical features is essential for both classification and regression tasks. Music emotion recognition (MER) is an interdisciplinary computational task [17], spanning fields such as psychology, neuroscience, and deep learning [7]. Currently, research on music affective recognition is primarily focused on two aspects: improving the accuracy of affective feature extraction for improved affective content expression and enhancing the accuracy and effectiveness of emotion recognition to better identify and understand affective cues in music.

Traditional features such as MFCC and average power were used as input features for music in [18,19,20,21,22]. In [23], the author selected force, range, time domain, and other variables as variables to extract music features, and trained a forward neural network (FNN) to improve the average recognition accuracy of the model to 86%. In addition, in order to better identify music affective information, it is necessary to construct a suitable network. In [15], an improved SVM algorithm was adopted for high-precision music classification. Hizlisoy et al. [24] constructed a hybrid structure based on LSTM and DNN, providing the energy and MFCC of logarithmic Mel filter banks to a convolutional neural network layer to obtain audio features. Zhong et al. [25] proposed a new network model, CBSA (CNN BiLSTM self-attention), in which a CNN is used to extract audio features, BiLSTM is used to capture temporal relationships between features, and a self-attention model is used to enhance the model’s representation ability.

Videos contain rich content and affective information, making research on video affective semantics significant. Currently, research on video affective analysis primarily revolves around refining methods for extracting affective feature. Traditional visual features primarily consist of color, texture, and region characteristics. With the advancement of deep learning technologies, these techniques are increasingly being utilized for the recognition and extraction of image emotional features [26,27,28,29,30,31,32]. Liao et al. [33] extracted two features, saliency target and facial target area, from an image and constructed corresponding deep learning frameworks to obtain their respective affective polarities. Lee et al. [34] first extracted relevant information about the facial region in an image, and then used attention mechanism based methods to extract relevant scene information from the remaining regions. Finally, they combined both types of information to recognize emotions. Zhan et al. [16] extracted three types of contextual information from images, including multimodal information, situational environment information, and depth map information, to complete affective recognition of image characters. This method can reduce dependence on facial expression information.

### 2.2. Audiovisual Association Matching

In the field of audiovisual cross-modal matching and retrieval, existing research has proposed methods such as label-based matching, self-supervised matching, and similarity-based matching. Label-based matching matches music and videos based on their label categories, considering a match when the categories align [14]. Self-supervised learning is a variant of unsupervised representation learning that exploits the intrinsic characteristics of unlabeled data as supervisory signals [35,36]. Surís et al. [37] proposed a self-supervised method that learns the direct correspondence between music and video from raw data, with artistic alignment of music and video serving as the supervisory signal for matching video segments with their corresponding music segments. Audiovisual matching methods based on similarity can be further divided into cognitive similarity and feature similarity. Cognitive similarity relies on human perception of emotions, such as using the valence–arousal (V-A) emotion model for emotional labels, and calculating the cross-modal similarity between music and video through cosine distance and Euclidean distance [17]. In contrast, feature similarity-based matching analyzes the associations between data at the feature level, with a typical approach being to learn shared representations of feature data from different modalities.

Currently, based on the cross-modal information used when learning shared representations, cross-modal retrieval can generally be categorized into retrieval and matching methods based on inter-data similarity and those based on inherent data correlations.

Based on inter-data similarity: Masci et al. [9] utilized a twin neural network to encode data from different modalities into binary codes, with performance superior to state-of-the-art hashing approaches. Cao et al. [38] proposed a deep hybrid structure called the correlation hashing network (CHN), which addresses the similarity issue of data in the shared representation space. Shen et al. [39] proposed a deep binary encoding method termed textual–visual deep binaries (TVDB) that is able to encode information-rich images and descriptive sentences. Liang et al. [40] expanded the traditional linear projection by utilizing affine transformation techniques, thereby constructing a universal similarity model. Nie et al. [41] proposed a novel deep hashing method (Dmfh) that can conduct feature learning and hash code learning simultaneously.

Based on inherent data correlations: Rasiwasia et al. [42] proposed canonical correlation analysis (CCA) to enable joint modeling of text and images. Andrew [43] introduced deep neural networks into the aforementioned concepts of CCA and defined deep CCA. Through the integration of DNNs, the linear mapping of data was substituted to achieve the maximization of data correlation. Shao et al. [44] employed a sequence of progressively enhanced DCCA models to incrementally improve feature representation and matching performance, thereby modeling the correlation between images and text. Zeng et al. [45] proposed a novel deep architecture composed of cluster-CCA and a deep triplet neural network model. This architecture fully considers the appropriate placement of each data point in the shared subspace based on pairwise correlations and semantic label assignments. Vukotic et al. [46] detailed a method for weight distribution and structural symmetry between autoencoders of different modalities to enhance the correspondence between data. Wei et al. [47] proposed an alternating optimization framework. This framework optimizes one modality’s autoencoder while keeping the weights of the other modalities’ autoencoders fixed, thereby enhancing the efficiency of cross-modal retrieval. He et al. [48] generated pairs of similar and dissimilar data in the latent space of the joint representation using maximum likelihood estimation and conducted comparative observations between the two. Yan [49] proposed a text-to-image retrieval system based on deep convolutional neural networks and utilized a margin-based objective function to constrain the similarity between data points. Zhang et al. [50] employed a combination of generative adversarial networks (GANs) and hashing functions to achieve unsupervised cross-modal hashing. Nawaz et al. [51] proposed a cross-modal retrieval method based on local features, which utilizes CNNs and RNNs to learn global and local features for each modality and employs a metric learning-based approach to achieve cross-modal matching. Gu et al. [52] employed multiple generators and discriminators to accomplish the tasks of image generation and discrimination. Su et al. [53] employed a bidirectional recurrent neural network to construct semantic relationships between images and text. Li et al. [54] introduced the cycle GAN framework to learn the joint representation of data in both real and binary values. Wang et al. [55] proposed a feature pseudo-labeling method named FPL. Integrating model predictions with feature similarity information enhances the robustness and accuracy of deep neural networks in the presence of label noise. Wang et al. [56] introduced a multimodal sentiment analysis method named CCDA, which integrates self-attention and cross-attention mechanisms. By incorporating a cross-correlation loss function, it effectively captures and fuses information across different modalities, significantly improving the model’s performance in multimodal sentiment analysis tasks.

The key to cross-modal prediction tasks is to find a suitable feature fusion method. Traditional early fusion methods amalgamate features from diverse modalities at the extraction stage, typically through concatenation or weighted summation [57]. Subspace-based techniques map disparate modal features into a common subspace for fusion at a higher-level representation [58]. Attention-based methods learn importance weights for different modalities [25]. Representation learning approaches aim to learn a shared subspace by optimizing their mutual correlation, yielding equivalent dimensional representations across all modalities [43].

Canonical correlation analysis (CCA) is considered a conventional representation learning method that takes the linear transformation of two modal datasets as input. However, this approach fails to guarantee the efficacy of feature extraction when variables exhibit nonlinear relationships. To address this issue, kernel canonical correlation analysis (KCCA) and deep canonical correlation analysis (DCCA) are employed to learn complex transformations of distinct datasets. In addition, cluster-CCA establishes all possible correspondences and utilizes CCA for projection learning, segregating different categories within the shared subspace to enhance intra-class similarity among data from various modalities [59].

KCCA utilizes kernel functions to map the original variables into a high-dimensional feature space, where potentially nonlinear relationships can be transformed into linear relationships [60]. The objective of KCCA is to find the optimal projection directions for two sets of variables in this high-dimensional space such that the correlation between the projected sets of variables is maximized. The core idea of this method is that even if the relationships between variables in the original data space are very complex, these relationships can be simplified and revealed in the new feature space through appropriate nonlinear transformations. Unlike CCA, KCCA is suitable for high-dimensional data and complex data relationships, as kernel methods can map data into a high-dimensional space without increasing computational overhead.

The core idea of DCCA is to project data from multiple modalities into a low-dimensional space, maximizing the correlation of the projected data in this lower dimension [41]. This method involves two deep neural networks, each dedicated to representation learning for data from the two modalities. In nonlinear feature mapping, DCCA exhibits superior performance, capable of maximizing the associativity of multimodal data and thereby addressing the issue of nonlinear transformation of input data. In contrast to CCA and KCCA, DCCA is tailored for temporal contexts, as it is capable of capturing the dynamic relationships between data points at various time points.

Cluster-CCA is a method employed when data can be categorized into distinct clusters or groups, facilitating the analysis of variable relationships between these clusters. By utilizing standard CCA for projection learning, it aims to maximize the correlation between two different modalities. Within the shared subspace, cluster-CCA seeks to isolate different categories, thereby enhancing the similarity of data from various modalities within each category. However, when it is applied to large datasets, the computational complexity is high and the processing efficiency is low. As with the above methods, this method is also suitable for small and medium-sized datasets. In the field of audiovisual retrieval, Zeng et al. [45] have achieved remarkable results by employing cluster-CCA for feature fusion, reaching an accuracy of 74.66% on the VEGAS dataset.

According to the above research, most existing algorithms measure cross-modal data in a common space for matching and retrieval. However, these methods may not finely capture the correspondence between different modalities, posing a challenge to effectively discover and capture valuable information.

To address the aforementioned issues, this paper integrates the cognitive and perceptual characteristics of emotions, constructing a matching model based on label similarity and feature fusion. In terms of feature fusion, this study used statistical methods to represent audio and video data as two multivariate datasets and established connections between them. Subsequently, the model utilized CCA and its variants to find the maximum correlation between these datasets, aiming to identify the greatest match between the two. On one hand, feature fusion can overcome the gap in cross-modal heterogeneity. On the other hand, constructing a factor space based on affective labels can fully explore the affective correlations across different modalities.

## 3. Proposed Methodology

In this work, we propose an audio and video matching model based on affective similarity and CCA. Thus, in Section 3.1, we establish the required music and video dataset. In Section 3.2, we propose a matching method based on affective similarity and CCA between the two modalities of music and video.

### 3.1. Music Video Affective Dataset

After reviewing relevant literature from domestic and international sources, it is evident that existing open datasets for cross-modal matching tasks between music and video do not meet the research needs for multidimensional affective semantics in this study. Specifically, these datasets can be categorized primarily into two distinct types. The first category is based on emotional similarity, which constructs music and video datasets respectively, annotated via continuous V-A spaces or discrete categories, such as the Music Video Emotion Dataset [61]. In these datasets, a match is considered to be established when the V-A values of music and video are close or when their categories align. The other category comprises self-supervised datasets, which are characterized by their use of original matched pairs as material, such as the Muvi dataset [62]. These datasets are designed to support binary judgments of uniqueness in retrieval tasks, meaning that the retrieval results are either a match with the original material or not a match at all. Indeed, in the task of cross-modal matching tasks between music and video, the degree of alignment can vary significantly. Seeking to explore matching relationships across different levels, we frame the task as a regression problem. Regrettably, the existing datasets are lacking in providing annotations for such multi-level matching.

Hence, to better reflect the diversity of matching, we consider constructing our own dataset. Specifically, the dataset material selection is based on videos and music in film scenes, where music and videos are closely related in affect, eliciting richer and deeper affective experiences. We have introduced manual matching, explicitly annotating the degree of alignment between music and video segments. The established matching pairs are roughly categorized into three types: original matches, which are the soundtracks of the videos, manually set matching data, and manually set non-matching data. The quantity of these three types of data is approximately equal. This design enhances the discriminability of our dataset in terms of matching degrees, thereby offering a clearer representation of varying levels of alignment. It also provides a basis for subsequent subjective evaluation annotation experiments, allowing for informed selection.

To clearly compare our dataset with other datasets, we have selected five existing datasets for comparison, as shown in Table 1. This study aims to explore the emotional alignment between movie and television videos and music, and hence our initial focus is on whether the dataset is derived from movie and television. Next, we consider whether the music and videos in the dataset contain a rich range of emotions.

#### 3.1.1. Dataset Establishment

Establishing a Movie and Television Music Dataset: For the required music dataset in this study, materials are balanced and selected based on four main points. Firstly, the scope of collection involves determining the background music sources and locations for selected video clips from film scenes. If a video lacks music, the theme song of the film is used as a replacement. Next is music genre diversity, encompassing various genres such as pop, classical, and rock music, along with music from different regions and languages, considering the quantities and proportions of each music type in the dataset. Another aspect is data quality, ensuring factors like recording quality, sound clarity, and sampling rate meet standards. Additionally, data integrity and consistency are crucial to maintaining dataset quality. Finally, affective categories’ balance directly affects the accuracy and reliability of subsequent affect analysis models. Collecting sufficient samples for each affect category ensures relative balance, ensuring the accuracy and reliability of affect analysis models.

In summary, we created 695 music clips based on the above factors, and the audio files were stored in WAV format.

Establishing a Movie and Television Video Dataset: For the required video dataset in this study, materials are balanced and selected based on two main points. Firstly, color balance in videos is crucial, as different colors evoke different emotional responses [49]. For instance, red symbolizes passion and excitement, blue represents calmness and serenity, while green signifies tranquility and nature. Due to people’s high sensitivity to colors, the dataset emphasizes balancing the main color tones in the visuals. The second point is the presence of characters in the scenes, as it is closely linked to affect. Characters provide additional information such as expressions, positions, postures, and genders. Clear and accurate facial expressions in images lead to higher accuracy in affect recognition. When characters are not visible, other visual features in the image, such as dark tones, blurred contours, and low saturation, can convey emotions. Hence, the dataset focuses on balancing the presence of characters in video scenes.

**Figure 1:**
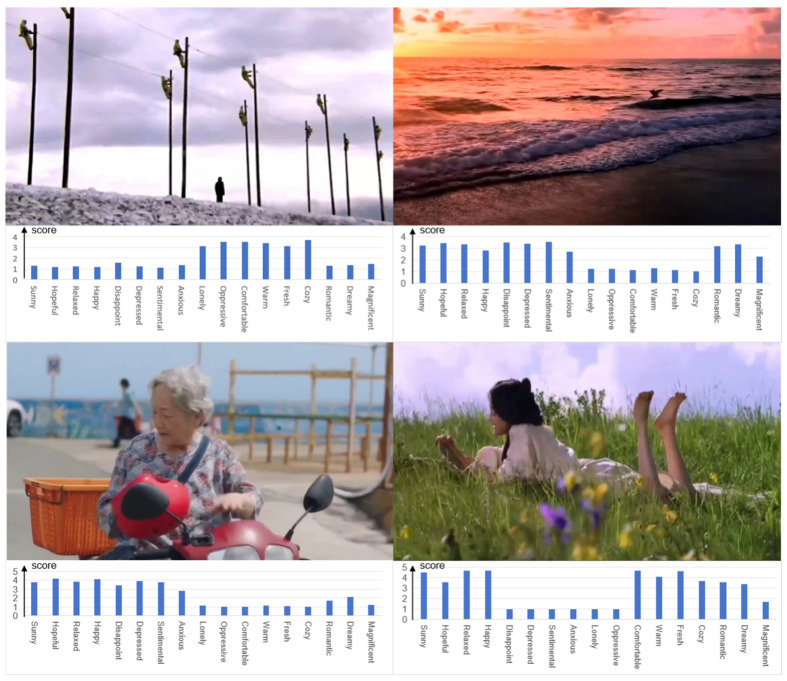
Example videos of the video dataset. The labels below the image represent the scores on a 5-point scale for 17 emotions.

In summary, this study created 300 video clips based on the above factors, with a storage format of mp4. An example of the video dataset is shown in Figure 1.

Establishing a Movie and Television Music Video Dataset: The affective model in this study aims to predict the level of affective matching between input audio and video. Therefore, based on the music and video datasets mentioned earlier, further work is needed to create music–video matching pairs by combining music and video visuals. Considering audiovisual quality, 980 music–video material combinations were selected for subsequent experiments, with 880 designated for the training set and the remaining 100 for testing.

#### 3.1.2. Affective Data Annotation

Subjective affect annotation experiments on the materials can study the emotions conveyed by different music and videos, quantifying affect using relevant evaluation metrics. The purpose of this experiment is to capture human subjective feelings towards different audiovisual materials and acquire affect parameters through subjective evaluation methods, laying the foundation for building an affective space. The affect parameters obtained in this section will be used for subsequent model construction, including building models based on CCA feature fusion and constructing an affective space based on factor analysis.

This paper adopts the five-level quantified scoring method of the Likert scale from social science research, combined with Su et al. [66]’s analysis of affective words in images, to determine 17 affect evaluation terms, forming the music–video affect evaluation system shown in Table 2. Additionally, a five-level quantified scoring method is used for annotating multidimensional affect and matching degree, as shown in Table 3.

According to the set of affective words and the scoring method given in the table, four groups of experiments were designed, as follows.

- Multidimensional affective annotation of video materials only: Participants are required to watch silent video clips in random order and use the affective evaluation system composed of multidimensional evaluation words to quantitatively describe the different dimensions of emotions in the video.
- Multidimensional affective annotation of music materials only: Participants are required to listen to music material in random order and quantitatively describe the different dimensions of emotions in the music using the affective evaluation system composed of multidimensional evaluation words.
- Multidimensional affective annotation of music–video material combinations: Participants were required to watch video clips with music in random order, and the affective evaluation system composed of multidimensional evaluation words was used to quantify the different dimensions of the combined emotion.
- Annotate the affective matching degree of music–video material combinations: Participants are required to watch video materials with music in random order and describe the degree of audiovisual affective matching for each combination of materials.

The collected experimental data were processed. Outliers that differed from the original mean by more than twice the standard deviation were removed and replaced with the original mean. The above data are used for the subsequent model construction based on CCA feature fusion.

**Figure 2:**
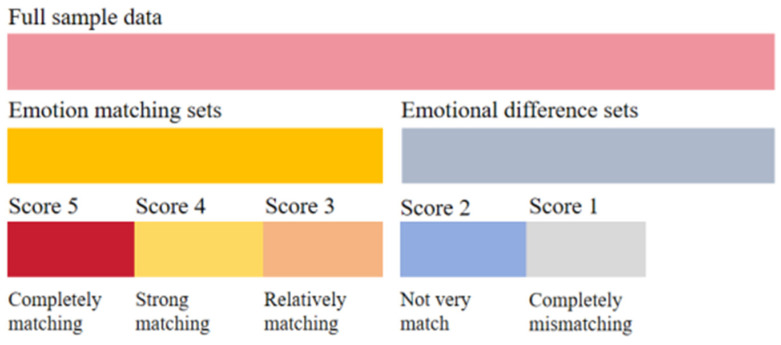
Sample partition set.

In order to deeply study whether there are differences between sets composed of samples with different affective consistency, the sample sets are further subdivided on the basis of Section 3.1.1. According to the labeling results in the matching degree experiment described in Section 3.1.2, the music–video pairs with scores 3 (relatively matching), 4 (strong matching) and 5 (completely matching) were divided into the affective matching sets, with a total of 553 entries. Similarly, the score 1 (completely mismatching) and score 2 (not very match) were divided into affective difference sets, totaling 347 items. Figure 2 illustrates the partitioning of the dataset.

### 3.2. Feature Extraction

#### 3.2.1. Music Stream

Given a music stream, both multidimensional statistical features and deep features of the music are extracted as input for the subsequent experimental model. Specifically, the MIRToolbox and TimbreToolbox are used to collect statistical values such as skewness, mean, and interquartile range. Ultimately, a 344-dimensional vector of traditional music features is obtained, including temporal, spectral, harmonic, rhythmic, timbral, tonal, and perceptual features. The VGGish model is used to extract 128-dimensional deep features. By combining traditional music features with deep music features, a combination of music feature vectors that are highly correlated with affective perception is achieved.

#### 3.2.2. Video Stream

Given a video stream, traditional and deep features are first extracted from the image frames of the video stream. Specifically, the first step is to use ffmpeg to decompose the video into frames and extract color images in jpg format. A 224-dimensional multi-level keyframe image feature set was extracted, including color, texture, composition, aesthetics, region, and other aspects [67]. Simultaneously, the extracted frames are fed into pre-trained feature extractors, including Vgg16 [68], InceptionV3 [69], C3D [70], MVIT [71] and TimeSformer [72]. The Vgg16 employed in this study is pre-trained on the ImageNet dataset and extracts 1024-dimensional deep features. InceptionV3 is also pre-trained on the ImageNet dataset and extracts 1000-dimensional image features. The C3D is pre-trained on the Sports-1M dataset and extracts 487-dimensional human motion features. TimeSformer and MVIT are pre-trained on the Kinetics dataset, extracting 400-dimensional and 1000-dimensional action features, respectively, that include temporal relationships. The mean squared errors (MSEs) of different video feature extractors for emotion prediction tasks are shown in Table 4. The best performance is indicated in bold. Therefore, Vgg16, which exhibits the best performance, is chosen as the video feature extractor. This indicates that the features within the image frames are important for fine-grained emotion. Thus, 1024-dimensional VGG16 deep video features are obtained. Finally, a 1248-dimensional feature vector is produced as the annotation feature of the video.

#### 3.2.3. Missing Value Processing Method

After the aforementioned stages of audio and video feature extraction, a small fraction of the data may still have missing values. Missing values refer to situations where certain data points are not fully collected or cannot be gathered during the data collection and organization process. For instance, during feature extraction, the presence of noise and outliers in the raw data might lead to the occurrence of missing values. Missing values may impact the outcomes of data analysis, necessitating their proper handling. Common methods for dealing with missing values include: deletion of missing values, interpolation, imputation of missing values, and model-based prediction.

Deletion of missing values involves the removal of rows or columns that contain missing data. This method may result in a significant loss of data, and thus it is only suitable when the proportion of missing values is relatively small. Interpolation involves using various interpolation techniques to predict missing values, such as linear interpolation, polynomial interpolation, and KNN interpolation. A limitation of this approach is that the predicted results might be influenced by the surrounding data. Consequently, when data are unevenly distributed or contain outliers, the outcomes of interpolation may not be optimal. Imputation of missing values involves filling in the missing entries with a specific value, such as the mean, median, or other statistical measures. This method is straightforward to implement. However, it may alter the distribution of the original data, potentially affecting the outcomes of subsequent analyses. Model-based prediction for handling missing values involves constructing models to estimate the missing data, such as using random forests or neural networks. These methods are capable of considering a large number of features, providing a more nuanced approach to imputing missing values.

Considering the analyses above, this paper utilizes random forest modeling to predict and impute the missing values. After traversing all features, the number of missing values is sorted in ascending order, and the feature with the fewest missing values is executed first, followed by the filling of missing values in the order of the sorted list. By constructing multiple decision trees to fill in missing values, this method better represents the actual distribution of these unknown data, making the imputed values more accurate and reliable, while also protecting the differences between various material segments.

### 3.3. The Audiovisual Matching Architecture

**Figure 3:**
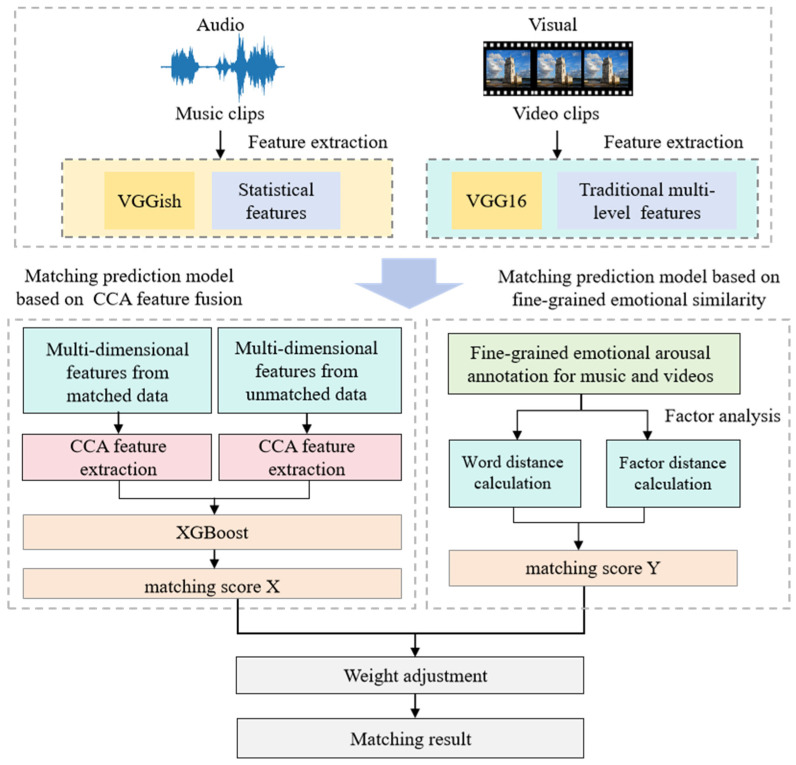
Flowchart of matching based on affective similarity and CCA feature fusion.

Considering the similarity and compatibility characteristics of video and music, we proposed a hybrid matching model based on affective similarity and CCA feature fusion. CCA feature correlation matching is introduced on the basis of factor space affective similarity, comprehensively considering the affective semantic data similarity between music videos and the internal relevance of the data, and combining feature and factor space layers to fully exploit the semantic relevance features of different modal data. The process of this method is shown in Figure 3.

On the one hand, it is necessary to calculate the matching score based on CCA feature fusion. Kernel canonical correlation analysis (KCCA) is utilized to project the cross-modal features of the two affective models into a shared feature space, where feature fusion is performed to effectively learn the nonlinear correlation of video–music data. Then, the new features obtained after KCCA are used as the input of XGBoost regression model to complete the final affective semantic matching degree prediction. From this, $X_{match}$ for the first part of the model was obtained.

Specifically, the process of feature fusion using KCCA is as follows. Consider $X_{v}$ is a combination of multidimensional feature vectors for video, and $X_{m}$ is a combination of multidimensional feature vectors for music. First, calculate the kernel function *k*, which is the inner product of video features and music features in high-dimensional space. ∅ represents the nonlinear mapping [60]. The formula for the kernel function is shown in (1).

(1) $$k(X_{vi},X_{mj})=\langle \emptyset (X_{vi}),\emptyset (X_{mj})\rangle$$

Hence, the kernel matrix is obtained, as illustrated in Equation (2).

(2) $$K=\left[k(X_{vi},X_{mj})\right]$$

Next, the regularized and centered kernel matrix is computed:

(3) $$\tilde{K}=JKJ+\eta I$$

where $J=I-\frac{1}{N}11^{T}$ denotes the centering matrix. I represents the identity matrix, 1 signifies a vector of all ones, and $11^{T}$ denotes the outer product of this vector. η is the regularization parameter, and *N* is the total number of samples.

By employing the kernel matrix $\tilde{K}$, the eigenvectors α and eigenvalues λ are determined, yielding the fused feature representation, as depicted in (4)–(6):

(4) $$\tilde{K\alpha }=\lambda \alpha$$

(5) $$Y_{v}=K\alpha$$

(6) $$Y_{m}=K^{T}\beta$$

where β is the eigenvector corresponding to α. $Y_{v}$ and $Y_{m}$ represent the projections of video and music in the new feature space, respectively. Subsequently, the correlation between $Y_{v}$ and $Y_{m}$ is computed in (7). The objective of KCCA is to maximize this correlation.

(7) $$\rho =\frac{\langle Y_{v},Y_{m}\rangle }{\left\|Y_{v}\right\|\left\|Y_{m}\right\|}$$

The aforementioned context illustrates the feature fusion process using KCCA. Indeed, as shown in the left part of Figure 3, this paper divides the dataset into two subsets, matched data and unmatched data. During the feature fusion process, feature fusion is performed within the matched data and unmatched data separately. Subsequently, the results are combined and used jointly as input for XGBoost to predict matching scores. This approach ensures the effectiveness of feature fusion to the greatest extent.

On the other hand, it is necessary to calculate the score based on fine-grained emotional similarity, which is calculated by integrating the sentiment evaluation word distance and the main factor distance. The word distance is calculated by applying the Euclidean distance of the farthest affective evaluation words between music and video, obtaining the affective word distance score $A_{w\_dis\_max}$ of the music and video. The factor space model inputs the 17-dimensional affective scoring data of music and video into the factor affective space model, obtaining the scores of four main factors on the factor affective space model. Then, the Euclidean distance is used to measure the farthest dimension factor to obtain $B_{f\_dis\_max}$. The similarity score $Y_{simi}$ of the second part of the model is obtained through (9).

Specifically, we choose factor analysis based on data dimensionality reduction, and further analyze the data results of the multidimensional affective labeling experiment in Section 3.1.2.

Word distance calculation: In emotion analysis, multidimensional affective word vectors can be used to represent the sentiment polarity of the material. Therefore, we adopt the word distance calculation method to evaluate the similarity between music and video in the multidimensional emotional space. In this method, affective scores will be calculated for each sample based on the 17-dimensional affective word evaluation results. Since each word is of equal importance in describing the affective space, Euclidean distance is introduced as a similarity measurement method [73]. The word distance formula is shown in Formula (8). $d_{w}(music,video)$ represents the total distance between music and video on the 17 affective words. $x_{music\_i}$ and $x_{video\_i}$ are the evaluation scores of music and video, respectively.

(8) $$d_{w}(music,video)=\sqrt{\sum_{i=1}^{17}{\left(x_{music\_i}-x_{video\_i}\right)}^{2}}$$

Factor distance calculation affective space model: The construction of the factor affective space model is based on the data obtained from the experiment in Section 3.1.2, firstly obtaining 17-dimensional sentiment scoring data for music and video materials, using SPSS to perform factor dimensionality reduction analysis on multidimensional affective words, and establishing our factor affective space model. Next, to ensure the practical significance of factor analysis, correlation analysis and Bartlett’s test are performed on the database. Typically, if the cumulative contribution rate of the first few factors is more than 85%, these factors can be considered to have good explanatory power for the variables. On this basis, the scores on the four main factor levels of music and video are obtained, and the Euclidean distance is used again to measure the similarity at the factor level to obtain the distance scores of the affective factors between the video and the music. The calculation method is shown in Formula (9). $d_{F}(music,video)$ represents the total distance between music and video for the four factors. $F_{music\_i}$ and $F_{video\_i}$ are the certain factors for music and video, respectively.

(9) $$d_{F}(music,video)=\sqrt{\sum_{i=1}^{4}{\left(F_{music\_i}-F_{video\_i}\right)}^{2}}$$

The factor affective space is shown in Table 5.

F1, F2, F3, and F4 in the table represent the four main scoring factors. The results of factor analysis show that the 17-dimensional affective words can be divided into four main factors, and all of them have good interpretability of emotional perception. Specifically, the first factor mainly reflects the affective representation of the material in terms of sadness. The second factor mainly reflects the affective representation of the material in terms of relief. The third factor mainly reflects the affective representation of materials in terms of “dreamy”, “romantic”, and “warm” atmospheres. The fourth factor is an affective representation of “magnificent”. Through the analysis and understanding of these factors, the affect of music and video can be better recognized so as to improve the accuracy and efficiency of emotion matching.

Matching method based on word distance and factor space: In order to make better use of multidimensional sentiment word distance and factor space distance information, a method of fully combining sentiment evaluation words and factor scores is adopted. This approach suggests that the Euclidean distance calculation of multidimensional sentiment words and the Euclidean distance calculation of the main factor scores in the factor affective space can be executed in parallel. By using the results of both for sentiment semantic matching, it can consider both the explanation of macro affects and the description of micro-semantics. The specific formula is shown in (10):

(10) $$Y_{simi}=\alpha \times A_{w_{-}dis_{-}max}+(1-\alpha )\times B_{f_{-}dis_{-}max}$$

where $A_{w\_dis\_max}$ is the distance between the two sentiment words with the farthest dimension in the process of calculating word distance. Similarly, $B_{f\_dis\_max}$ represents the distance between the two factors in the farthest dimension when calculating the factor distance between music and video. Obviously, $A_{w\_dis\_max}$ and $B_{f\_dis\_max}$ both represent a measure of similarity, used to measure similarity at different levels. Then, assign different weights to $A_{w\_dis\_max}$ and $B_{f\_dis\_max}$ and combine them to obtain the final affective matching distance. The establishment of weight α is introduced in Section 4.

Building upon the aforementioned, this paper constructs a hybrid matching model based on affective similarity and CCA feature fusion that comprehensively considers the similarity between affective semantic data in music videos and intra-data correlations. By integrating both the feature-level and factor space aspects, this model extensively explores the semantically correlated features of different modal data. It is suggested that matching based on CCA feature fusion and matching based on factor relationship similarity can proceed in parallel, each being assigned different weights to achieve the optimal predictive performance, as shown in (11):

(11) $$M_{score\;}=\beta \times X_{match\;}+(1-\beta )\times Y_{simi\;}$$

where $M_{score}$ is the affective matching score between music and video. The determination of weight β will also be introduced in Section 4.

## 4. Experiments and Results

In this section, we conduct feature analysis (in Section 4.1), parameter determination (in Section 4.2), comparison with other methods (in Section 4.3), and discuss the results (in Section 4.4). When training the affective matching prediction model, it is necessary to select appropriate evaluation metrics for regression problems to measure the performance of the model. The mean square error (MSE) is selected as the evaluation index in this experiment. The MSE value is easy to calculate and interpret, providing an intuitive reflection of the model’s predictive accuracy. The formula is as follows:

(12) $$\mathrm{MSE}=\frac{1}{m}\sum_{i=1}^{m}{\left(y_{i}-{\hat{y}}_{i}\right)}^{2}$$

Based on the construction of the above model and the selection of evaluation indicators, we conduct analysis experiments. All experiments in this paper were implemented in Matlab and the hardware environment was a computer with Intel(R) Core(TM) i7-10510U CPU @ 1.80 GHz, 2.30 GHz, processor and 16 GB memory. The software environment operating system is Windows 10 64 bit.

### 4.1. Subsets Analysis

During the experiments, in order to avoid redundant features occupying memory, we performed single-modal internal dimensionality reduction on audio and video features. Principal component analysis (PCA) [74], singular value decomposition (SVD) [75], and random forest (RF) [76] algorithms were used for dimensionality reduction, and the model performance was compared with the original feature dimension.

For the selection of the learner, this paper aims to predict matching scores between music and video clips, which falls under the category of a regression problem. Boosting is an ensemble learning technique whose fundamental approach involves sequentially training a series of weak learners to construct a strong learner. Compared to other machine learning methods, boosting algorithms have advantages in terms of accuracy and scalability. For the sake of training efficiency, we chose LightGBM [77] and XGBoost [78] from the boosting method for subsequent experimental analysis.

LightGBM is a gradient boosting decision tree (GBDT) model that efficiently enhances training speed and accuracy through an innovative histogram algorithm. This algorithm significantly reduces computational complexity and memory usage by discretizing feature values into a fixed number of bins, effectively mitigating the risk of overfitting. XGBoost employs the gradient boosting algorithm to construct an ensemble model, where each iteration adds a new decision tree to correct the residuals from the previous round. In this way, XGBoost can effectively reduce the error and improve the accuracy. Both of the two models are suitable for large-scale datasets and are capable of handling complex, high-dimensional data.

This step still used MSE as the performance evaluation indicator. The results are shown in Table 6.

**Figure 4:**
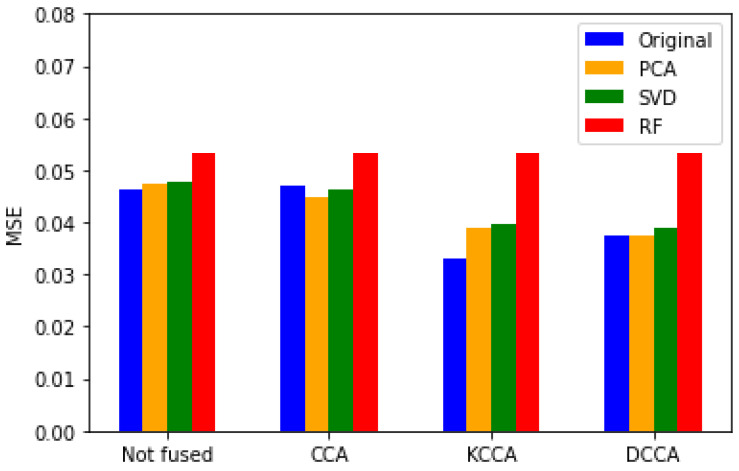
Comparison of MSE using different feature fusion methods.

From the results in Table 6, it can be observed that in terms of affective matching model performance, LightGBM demonstrates superior overall predictive effectiveness to XGBoost, attributable to LightGBM’s use of techniques like GOSS and EFB to enhance accuracy. However, when using KCCA and XGBoost, the model performs optimally, with the best MSE value being 0.0331. This is due to XGBoost’s gradient boosting framework combined with KCCA, enabling better data fitting and improving predictive accuracy. The outcomes of different feature fusion methods based on XGBoost are illustrated in Figure 4.

As depicted in Figure 4, concerning intra-modal dimensionality reduction, the best results are obtained when not reducing the original feature dataset. This is because using the original features can prevent information loss, whereas dimensionality reduction may lead to the loss of crucial information essential for model training and prediction capabilities. Directly modeling with the original data can better preserve the original structure of the data.

Regarding cross-modal feature fusion, overall, the results show that the use of CCA and its variant methods outperforms not fusing cross-modal features. This indicates that the fusion of features at the feature level using CCA-based methods yields good results. By leveraging pair information from different modal samples for learning common subspaces and measuring the similarity between different modal data within the subspace, it helps improve model prediction accuracy. Comparative analysis of several CCA methods reveals that the performance of KCCA feature fusion is significantly better than traditional CCA and DCCA. This is attributed to KCCA’s ability to capture correlations among data better than traditional CCA and DCCA, learning more complex nonlinear relationships, avoiding overfitting issues, and exhibiting more stable performance. In cases of small-scale datasets, DCCA may not fully exploit the strong representation learning capabilities of deep neural networks, hence not performing as well as KCCA. Traditional CCA lags behind DCCA, as traditional CCA may directly identify the direction of maximum correlation. However, due to its strict assumption of linear relationships among data, traditional CCA may struggle when handling complex data relationships, leading to relatively poor performance.

Further research was conducted on the two subsets for the sub-affective model features. KCCA was used for cross-modal feature fusion, and the XGBoost model was used to complete the correlation regression prediction. In this step, MSE was still selected as the effect evaluation index. The relevant results are shown in Table 7.

It can be seen in Table 7 that the MSE of both sub-models is higher than 0.0331, indicating inferior performance compared to the hybrid matching model. This suggests an overall improvement in performance with the hybrid matching model, confirming the rationality behind matching based on sub-affective models.

### 4.2. Parameter Determination

Determination of α in the factor model: In this paper, distance accuracy is used to measure the experimental results and complete the determination of the parameter α. Distance accuracy is defined as the ratio of the number of predicted results whose distance between the final predicted result and the subjective experimental value is below the average distance of the sample set to the total number of predicted results, and the calculation formula is shown in (13).

(13) $$Distance\;accuracy=\frac{The\;number\;of\;predicted\;distances\;below\;the\;average\;distance}{The\;number\;of\;overall\;predictions}\times 100\%$$

Apparently, the larger the value, the better. When the distance accuracy is higher, it means that the number of samples whose distance between the predicted value and the subjective experiment is below the average distance level of the sample set is larger, implying more accurate predictions. The experimental results are shown in Table 8, where N is the number of predicted distances below the average distance. The bold numbers indicate the best results at that metric.

When α=0.6, the distance accuracy is the highest. This means that in the process of combining the two kinds of affective distances, the distance of the farthest dimensional affective words in the 17-dimensional affective words is more important to the final output. However, the distance of the farthest dimension factor among the four main factors has a weak influence on the final output score. This is due to the fact that information is not extracted during factor analysis, resulting in information waste.

Determination of β in the hybrid model: Firstly, the prediction result of the matching model based on CCA feature fusion $X_{match}$ was obtained, the weight β was given, and the weighted score β × $X_{match}$ was calculated. Then, the prediction score of the matching model based on affective similarity $Y_{simi}$ was obtained, the weight (1 − β) was given, and the weighted score (1 − β) × $Y_{simi}$ was calculated. The results of the two parts are summed, and the mixed prediction value is obtained for each music–video pair as the final prediction score of the material, and then the distance between the mixed prediction value and the subjective experimental value is calculated. The above operation was performed on 880 sample pairs to obtain the total distance of the sample set, and the average distance level of the sample set under this method was further obtained. At the same time, the number of sample pairs below the average distance level is accumulated, and the distance accuracy under different β values is obtained by (11). The experimental results are shown in Table 9, where N is the number of predicted distances below the average distance. The bold numbers indicate the best results at that metric.

According to Table 9, when β is 0.7, the matching method proposed in this paper has the highest accuracy. This suggests that in the process of combining the two matching models, the impact of the CCA feature fusion matching model on the final predictive accuracy is more significant. Meanwhile, the performance of the prediction model based on factor relationship similarity is relatively weaker, considering that similarity-based cross-modal matching methods typically utilize only some information from each modality, resulting in information loss. Based on the above analysis, the parameter β of our proposed model approach is determined to be 0.7.

### 4.3. Comparison with Other Methods

The hybrid matching model with the determined parameters is applied to the test set. The results show that the MSE value of the proposed affective audio–video hybrid matching model is 0.029947.

**Figure 5:**
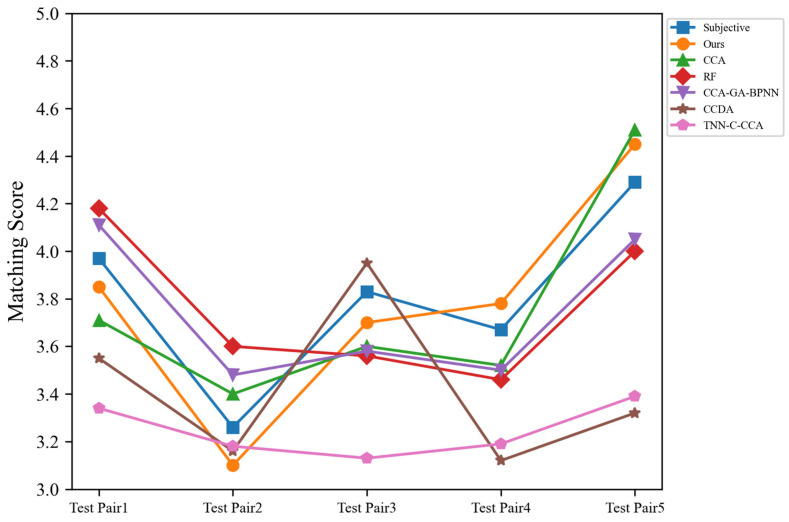
The predicted values of several methods are compared with the subjective experimental values, where the horizontal coordinate of the chart is the serial number of the test pair, and the vertical coordinate is the affective matching score. The 5 test pairs are drawn from the test set of representative scenarios. “Subjective” refers to the matching scores obtained through subjective evaluation experiments.

In order to better illustrate the advantages of the proposed matching model, it is compared with the CCA-GA-BPNN [79] method and the random forest (RF) [80]-based method, and the MSE results of the affective matching degree of relevant music–video pairs are shown in Table 10. Among them, the CCA-GA-BPNN [79] model employs CCA for data dimensionality reduction and utilizes a genetic algorithm to optimize the weights and thresholds of the neural network structure. The emotion matching based on the random forest (RF) algorithm [80] primarily extracts a total of 64-dimensional music features, including MFCC, centroid, roll-off, and flux features, and completes the model training through the random forest algorithm. CCA feature fusion [42] employs CCA to integrate cross-modal features and then uses XGBoost for prediction. The CCDA [56] model employs self-attention and cross-attention mechanisms to fuse features and is optimized using a cross-correlation loss. The method based on TNN-C-CCA [45] utilizes VGGish to extract audio features and Inception to extract video features. The model employs cluster-CCA for feature fusion and predicts through a deep triple neural network. To compare the performance of the algorithm presented in this paper, we have modified the classification tasks in relevant literature into regression tasks. For some of the networks originally used for audiovisual retrieval, we retained the form of their backbone networks and adjusted the loss functions. All experiments were conducted on our own dataset. The comparison between the predicted values of several methods and the subjective evaluation values is shown in Figure 5. In order to facilitate observation, parts of the results are selected, and the subjective experimental values are used as the basis for discrimination. The bold numbers indicate the model with best results at that metric.

As can be seen from the results in Table 10, the MSE values of the five comparison methods on the dataset in this paper are significantly higher than those of the model proposed in this paper, indicating that the prediction effect of the literature method is not ideal. However, the hybrid matching model based on affective similarity and CCA feature fusion proposed in this paper has better performance.

It can be intuitively found from Figure 5 that the result of the hybrid matching model based on CCA and factor space proposed in this paper is the best, indicating that its prediction effect is most similar to the subjective emotion of humans.

### 4.4. Experimental Results

This paper aims to predict the matching degree between music and video by fusing affective similarity and CCA features. In this paper, the prediction model with the best MSE score is selected as the sentiment prediction value of the hybrid matching model, and the relevant experimental results are shown in Table 11. When users input a music clip and a video clip into the proposed model for affective prediction, the model will automatically calculate and return a matching score based on the input data. This score can be seen as a metric to measure the degree of affective matching between audio and video, and it can help users better understand the affective association between music and video.

## 5. Conclusions

To address the demand for intelligent understanding and matching of video and music in film and television scenes, we proposed a hybrid matching model based on affective similarity and CCA feature fusion. The model enables end-to-end audiovisual matching computation and retrieval, considering the perceptual and cognitive fusion of cross-modal elements, leading to more intuitive and accurate matching results that correspond with human subjective aesthetic preferences.

Human perception of audiovisual content is a complex process. Although artificial intelligence and deep learning technologies can assist in better identifying the intrinsic affects within artistic works, a deeper understanding of the underlying association mechanisms requires further experiments and exploration. For instance, in the analysis of extended videos, matching may not be confined to the overall stylistic unity, but could involve the aspects of camera movements, image transitions, or rhythmical points. Also, the dominant matching factors in different types of video and music might be certain affective distributions across the special modalities, while the existing methods have not yet fully addressed these problems. Therefore, work will continue to evolve following these new research avenues, with an intention to integrate the algorithm of this paper into sensors to construct a perceptual system.

## Figures and Tables

**Table 1:** Emotional music and video datasets. Within the table, the symbol “√” denotes “yes”, while “—” denotes “no”.

Dataset	From Movie and Television	Open Source	Size	Emotional Labels	Divide between Matched and Unmatched Pairs
AFEW [63]	√	√	1426 clips from 0.3 to 5.4 s long	7 categories	—
Music Video Emotion Dataset [61]	—	√	1699 clips from 25 to 35 s long	6 categories	—
Muvi [62]	—	√	81 one-minute videos	V-A; GEMS28	—
LIRIS-ACCEDE [64]	√	—	9800 excerpts from 8 to 12 s long	PAD	—
MVD [65]	—	√	1600 ids from YouTube videos	—	—
Our Dataset	√	—	980 clips from 5 to 15 s long	Fine-grained emotion	√

**Table 2:** Affective evaluation word set.

Sentiment	Sentiment
Sentimental	Warm
Depressed	Sunny
Disappoint	Cozy
Anxious	Happy
Oppressive	Hopeful
Lonely	Relaxed
Romantic	Dreamy
Magnificent	Fresh
Comfortable	

**Table 3:** 5-level quantitative scoring method.

Score	Name of Experiment	
	Affective Labeling	Matching Degree Labeling
1	No at all	Completely mismatching
2	Not too strong	Not very match
3	General	Relatively matching
4	Strong	Strong matching
5	Very strong	Completely matching

**Table 4:** Prediction results based on different video feature selection methods. The lower the MSE, the stronger the performance of the model.

Model	MSE
C3D [70]	0.0409
MVIT [71]	0.0506
TimeSformer [72]	0.0347
InceptionV3 [69]	0.0766
Vgg16 [68]	**0.0252**

**Table 5:** Factor affective space model.

Sentiment	Music Video Composition Components (Factors)			
	F1	F2	F3	F4
Sunny	−0.704			
Hopeful	−0.668			
Relaxed	−0.618			
Happy	−0.773			
Disappoint	0.905			
Depressed	0.867			
Sentimental	0.946			
Anxious	0.820			
Lonely	0.954			
Oppressive	0.660			
Comfortable		0.706		
Warm		0.592		
Fresh		0.660		
Cozy			0.708	
Romantic			0.797	
Dreamy			0.914	
Magnificent				0.960

**Table 6:** Performance comparison of music–video matching models based on CCA analysis. The lower the MSE, the stronger the performance of the model. Bold numbers indicate the model with the best results at that metric.

Model	Feature Fusion	MSE			
		Original Dimension	PCA [74]	SVD [75]	RF [76]
XGBoost [78]	\	0.0463	0.0473	0.0479	0.0532
	CCA [42]	0.0472	0.0448	0.0464	0.0532
	KCCA [60]	**0.** **0331**	0.0390	0.0398	0.0533
	DCCA [43]	0.0376	0.0374	0.0390	0.0532
LightGBM [77]	\	0.0443	0.0472	0.0468	0.0533
	CCA [42]	0.0458	0.0474	0.0465	0.0533
	KCCA [60]	0.0342	0.0410	0.0406	0.0532
	DCCA [43]	0.0371	0.0363	0.0390	0.0533

**Table 7:** Comparison of affective matching model and affective difference model.

Sub-Model	MSE
Affective matching model	0.0378
Affective difference model	0.0365

**Table 8:** Distance accuracy corresponding to different α of the factor sentiment space model.

Metrics	Value of α										
	0	0.1	0.2	0.3	0.4	0.5	0.6	0.7	0.8	0.9	1.0
N	444	452	459	447	440	466	**478**	472	470	463	463
Accuracy (%)	50.5	51.4	52.2	50.8	50.0	53.0	**54.3**	53.6	53.4	52.6	52.6

**Table 9:** Accuracy corresponding to different β values of the mixture model.

Metrics	Value of β										
	0	0.1	0.2	0.3	0.4	0.5	0.6	0.7	0.8	0.9	1.0
N	376	485	460	447	440	452	494	**515**	463	447	424
Accuracy (%)	42.8	55.1	52.3	50.8	50.0	51.4	56.1	**58.5**	52.6	50.8	48.2

**Table 10:** Performance comparison of several models.

Methods	MSE
CCA-GA-BPNN [79]	0.0431
RF [80]	0.0523
CCA feature fusion [42]	0.0304
Modified CCDA [56]	0.0349
Modified TNN-C-CCA [45]	0.0388
Affective similarity and CCA feature fusion (ours)	**0.0299**

**Table 11:** Partial experimental results of the hybrid matching method.

Video Clips	Music Clips	Affective Matching Values	
		Subjective Value	Predicted Value
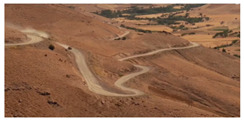	Little Astronaut Henry Lai	3.42	3.33
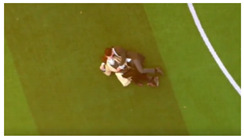	The First Words Song Yu Vin	3.13	3.00
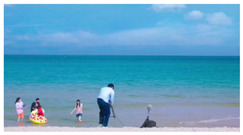	Let the World be Filled with Love Various Artists	4.24	4.42
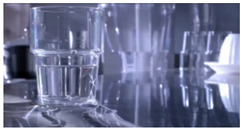	Water Of Life Silver Screen	4.07	4.18
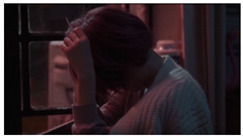	Because I Only See You Kim Nayoung	3.90	3.82

## Data Availability

The data that support the findings of this study are available from the corresponding author upon reasonable request. The data are not publicly available due to privacy or ethical restrictions.
